# Optimizing Lactic Acid Bacteria Fermentation for Enhanced Summer and Autumn Tea Quality

**DOI:** 10.3390/foods13193126

**Published:** 2024-09-30

**Authors:** Xiaoli Mo, Yingyu Chen, Zhen Zeng, Sui Xiao, Yahui Huang

**Affiliations:** 1 College of Horticulture, South China Agricultural University, Guangzhou 510642, China; mxiaol1997@163.com (X.M.); zzhen2004@126.com (Z.Z.); 2 Tea Research Institute of Hunan Academy of Agricultural Sciences, Changsha 410125, China; chenyy2405@163.com; 3 College of Forestry and Landscape Architecture, South China Agricultural University, Guangzhou 510642, China; xiaosui@scau.edu.cn

**Keywords:** lactic acid bacteria, tea fermentation, process optimization, GABA

## Abstract

The level of consumption of summer tea is a problem in the development of China’s tea industry. Current strategies to enhance the quality of summer and autumn teas primarily target the cultivation environment, with less emphasis on processing improvements. This study aimed to optimize the fermentation parameters to impact the quality of summer and autumn teas. We screened four strains of lactic acid bacteria (LAB) suitable for tea fermentation and determined their optimal mix. This optimized blend was applied to ferment summer and autumn teas. Through single-factor experiments, we evaluated the impact of various processing parameters, including the fixation method, rolling degree, inoculation amount, glucose concentration, fermentation temperature, and fermentation duration, on LAB growth and tea quality. The optimal processing conditions were established as microwave fixation, heavy rolling, an inoculation rate of 1.8% LAB, glucose addition at 8.8%, and fermentation at 36.5 °C for five days. Analysis revealed that the fermentation process significantly reduced the levels of polyphenols and ester-type catechins, which are associated with astringency and bitterness while enhancing the content of gamma-aminobutyric acid (GABA). Specifically, after five days, polyphenol content decreased by 26.89%, and GABA levels increased from 0.051 mg/g to 0.126 mg/g. The predominant aroma compounds in the fermented tea were alcohols with floral and fruity scents, constituting 54.63% of the total aroma profile. This research presents a methodical approach to reduce the astringency and bitterness of summer and autumn teas while concurrently increasing GABA levels.

## 1. Introduction

Tea is one of the top three non-alcoholic beverages in the world and is popular among consumers for its rich variety and health benefits [1,2,3,4]. Chinese tea is rich in categories, which can be divided into green tea, white tea, yellow tea, oolong tea, black tea, and dark tea, according to the degree of fermentation [5]. Green tea is the only non-fermented tea among these six tea types, and its processing course is fresh leaves → withering → fixing → rolling → drying. Fresh leaves are promptly fixed after picking, so they contain more polyphenols and a higher original aroma than fermented tea and retain better flavor and biological activity [6,7].

In tea consumption, people prefer spring tea because the light, temperature, humidity and other conditions in spring are suitable for the growth of tea trees, and higher quality tea can be obtained with fresh tea leaves [8]. Tea plants are shade-tolerant and thrive best in diffuse light. The higher temperature and strong light in summer and autumn cause polyphenol content in tea leaves to increase and free amino acid content to reduce, resulting in astringent tea soup [9]. Hence, the fresh leaves of summer remain almost unpicked. The low utilization rate of summer tea is still one of the problems in the development of China’s tea industry [10]. The methods to improve the quality of summer and autumn tea are mostly focused on the growing environment of fresh tea leaves, with shade being used to reduce the generation of polyphenols and other compounds [11,12], whereas less are focused on the processing point of view.

Fermentation is an important process in tea processing, except for green tea. There are two types of tea fermentation. One involves an enzymatic oxidation reaction [13], which is applied in the production of black tea. The other relies on microorganisms for fermentation. Dark tea is mainly fermented by mold, yeast, and certain bacteria. The dominant microorganisms in the fermentation process of dark tea vary from one type to another [14,15,16]. After a variety of microbial fermentations, the contents of the tea leaf have changed, and many microbial metabolic active substances have also been brought in to improve the nutritional value of the tea [17]. Lactic acid bacteria (LAB) have health functions such as antioxidant, reduction of uric acid synthesis, and preservative effect of egg products and are widely used in food, medicine and health care, agriculture, and livestock disease control [18]. LAB are also a common microorganism for tea beverage development, and it was found that lacto-fermentation can significantly increase the amino acid content in tea beverages [19], and the finished beverage has a mellow taste with both tea and milk flavors [20].

The main purpose of this study was to investigate the effect of LAB fermentation on the quality of summer-autumn green tea and to explore ways to reduce the bitterness and astringency of summer-autumn tea soup while increasing the active ingredients in tea leaves and improving the utilization and quality of summer-autumn tea.

## 2. Materials and Methods

### 2.1. Materials and Drugs

The fresh tea leaves used for making LAB fermented tea samples were one bud with two or three leaves of *Camellia sinensis* cv. Fuyun 6, which were all collected from the SCAU Main Campus Teaching & Research Base during the summer.

Of six strains of lactic acid bacteria (LAB) used in the experiment, three were from the laboratory of the College of Food Science of SCAU, namely *Lactobacillus plantarum* (*L. plantarum*), *Streptococcus thermophilus* (*S. thermophilus*), and *Leuconostoc mesenteroides* (*L. mesenteroides*); the others were purchased from Weike Biotechnological Co., Ltd. (Shanghai, China), namely *Streptococcus acidophilus* (*S. acidophilus*), *Lactobacillus brevis* (*L. brevis*), and *Lactobacillus bulgaricus* (*L. bulgaricus*).

Methanol and forintol (AR grade) were used for tea polyphenol detection.

Disodium hydrogen phosphate dodecahydrate (AR grade), potassium dihydrogen phosphate (AR grade), and ninhydrin (AR grade) were used to determine free amino acids.

All drugs in the AR grade were purchased from Macklin Biotechnological Co., Ltd. (Shanghai, China).

Gallic acid (GA), (-)-epicatechin (EC), (-)-epigallocatechin (EGC), Catechin (C), (-)-epigallocatechin-3-gallate (EGCG), and (-)-epigallocatechin (ECG) (HPLC) were purchased from Shyuanye Biotechnological (Shanghai, China) Co., Ltd.; trifluoroacetic acid (HPLC) was purchased from Sangon Biotech (Shanghai, China) Co., Ltd.; methanol (HPLC) was purchased from Thermo Fisher Scientific Inc. (Newton Drive, Carlsbad, CA, USA) and used as the solvent.

### 2.2. Methods

#### 2.2.1. Determination of LAB Fermentation Strains

Strain activation and viability determination: The frozen-preserved strains were inoculated into MRS liquid medium and incubated at 32 °C and 120 r/min in a shaking incubator for 48 h. The activated LAB were inoculated into MRS liquid medium at 2% inoculum and incubated at 32 °C; samples were taken every two hours, and the OD was measured at 600 nm. For the acid production capacity assessment, an aliquot of the culture was incubated under the same conditions, and the process was allowed to proceed for a total of 48 h. At 4-h intervals, samples were collected to measure the pH level, indicating acid production. This experiment was conducted in triplicate to ensure the reliability of the results.

Determination of the fermentation strains: Six strains of LAB were inoculated into tea leaves at 2% inoculum and incubated at 32 °C for five days, and the number of active bacteria and lactic acid yield in the fermentation system was measured. Each treatment was repeated three times, and the four strains with better fermentation performance were finally selected as fermentation strains for subsequent tests. The inoculum was specified to be 2%, and the four strains of LAB selected were mixed for fermentation. The lactic acid yield and active bacteria counts in the fermentation system were used as response values, and the data were analyzed using Design–Expert (v.12, NCSS, LLC, Kaysville, UT, USA) so as to construct model equations and predict the best fermentation strain ratios. The factors and levels of the LAB mixing test are shown in Table S1.

#### 2.2.2. Single-Factor Tests of the LAB-Fermented-Tea Process

Fermented tea sample production process: picking fresh leaves → fixing → rolling → bottling → inoculation → fermentation → drying. The level design of single-factor tests of the fermentation process is shown in Table S2. After the fermentation was completed, the tea leaves were dried and crushed with a pulverizer and passed through a 40-mesh sieve to determine the biochemical composition of the tea leaves. All experiments, including processing and analytical tests, were repeated three times.

Based on the predicted results of Section 2.2.1, the mixed-bacteria fermentation was carried out, and the fermented-tea process was determined by single-factor tests, with lactic acid yield as the measurement index. The tests were repeated three times. Tables S3 and S4 show the factors and levels of the mixing test.

#### 2.2.3. Determination Method of Test Index

Determination of active bacteria counts: The flat colony counting method with solid MRS medium was used and incubated at 37 °C for 48 h. The determination of total acidity was performed following the protocol described by Avdeef et al. [21].

The moisture and tea polyphenol contents were determined using the methods referenced by Guo et al. [22]. The amino acid content was analyzed following the protocol by Fan et al. [23]. The determination of the catechin component was carried out by high-performance liquid chromatography (HPLC) established by the group in the early stage [24].

GC-MS was applied to determine the aroma substances, and the determination method has been improved upon the foundation laid by previous researchers [25]. Sample preparation: 0.5 g of tea sample was taken, ground, placed in a reagent bottle, added with 2.25 mL of dichloromethane and 5 nmol of ethyl aconitate (internal standard), and extracted by shaking at room temperature for 6 h. Then, the extracted sample was dried with anhydrous sodium sulfate, added to the injection bottle, properly placed in the auto-sampler, and prepared for analysis and processing by the GC-MS coupler. Chromatographic conditions: DB-5MS flexible quartz capillary column (30 cm × 0.25 mm, 0.25 μm); inlet temperature, 230 °C; flow rate, 1.0 mL/min; no split injection; carrier gas, He. Ramp-up procedure: keep at 60 °C for 3 min, ramp up to 240 °C at 4 °C/min, lasting for 20 min, column chamber temperature, 60 °C. Electron ionization source: electron energy, 70 eV; ion source temperature, 230 °C; MS interface temperature, 250 °C; solvent delay time, 9.5 min; scan start time, 10 min; end time, 60 min.

## 3. Results and Discussion

### 3.1. Results of LAB Screening

**Figure 1:**
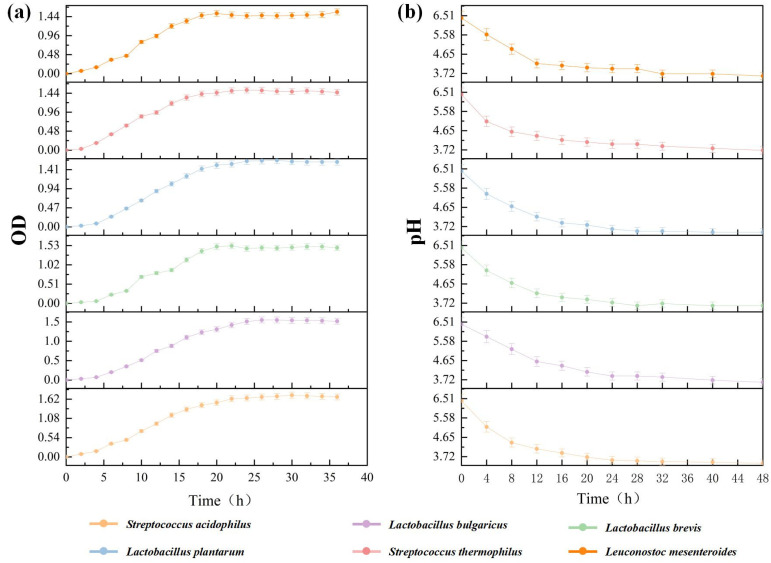
Growth curve and pH value change of six strains. Note: (**a**) Growth curves of six strains; (**b**) Lactic acid bacteria liquid culture medium pH changes over time.

Different strains have their own suitable growth environment and fermentation characteristics. Therefore, the detection of various physiological characteristics of the strains before fermentation can provide a basis for selecting LAB for food fermentation and screening LAB with good adaptability in the fermentation substrate, thereby ensuring the quality of fermented food and improving production efficiency. *Streptococcus acidophilus* has the strongest growth ability, and *L. bulgaricus* has the weakest (Figure 1a). At 22–24 h of fermentation, the LAB hardly continues to grow, and the light absorption value remains unchanged. In order to improve the efficiency of the test operation, all the strains selected to inoculate were cultivated for 20 h. At this point, the growth state of all LAB was in the geometric logarithmic growth interval, and they could be rapidly expanded after inoculation. The acid production performance of *L. bulgaricus* and *S. thermophilus* was similar and poorer among the six strains of LAB. At 48 h, the pH value of the *S. acidophilus* medium dropped to 3.42, while that of *L. bulgaricus* and *S. thermophilus* were 3.88 and 3.73, respectively, which were significantly larger than *S. acidophilus*. The acid production performance of the other three strains of LAB was between *S. acidophilus* and *L. bulgaricus*. The pH change curve of *L. brevis* overlapped mostly with that of *L. plantarum*, and the acid production performance of the two was similar (Figure 1b).

All six strains of LAB were able to grow and produce acid normally after inoculation into tea leaves, and the active bacteria counts reached 10^7^ cfu/mL or more after 24 h; the amount of lactic acid produced was more than 5 g/L. The fermentation effect of *S. acidophilus* in tea leaves was the best, and the active bacteria counts reached 7.84 cfu/mL; the amount of lactic acid was 7.04 mg/g after 24 h (Table 1); *L. plantarum* and *L. mesenteroides* grew well in tea with higher acid production of 6.54 mg/g and 6.78 mg/g, respectively; *L. bulgaricus* as well as *S. thermophilus* differed from the other four LAB by one order of magnitude in terms of active bacteria count.

The adaptability of the six strains of *Lactobacillus* in tea growth and acid production performance were combined, and *S. acidophilus*, *L. brevis*, *L. plantarum,* and *L. mesenteroides* were selected as tea blend fermentation strains.

### 3.2. Determination of the Proportion of Bacteria in Mixed Fermentation

**Figure 2:**
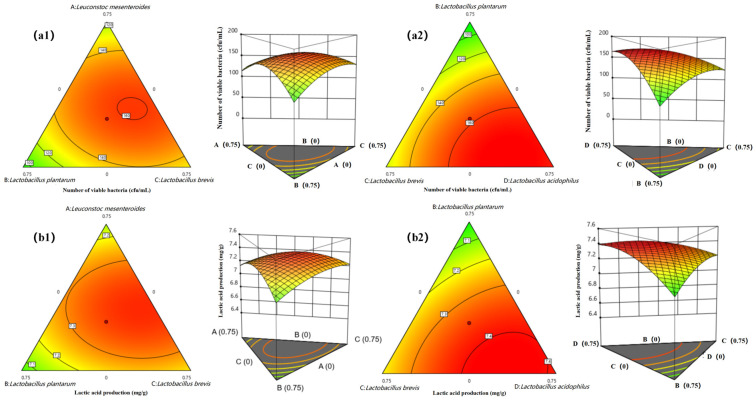
Effect of the interactions between strains on viable bacterial count and the lactic acid production. (**a1**,**a2**) show the effect of the interaction between *Leuconostoc mesenteroides*, *Lactobacillus plantarum,* and *Lactobacillus brevis* on the viable bacterial number and the lactic acid production of the fermentation system when the inoculum of *Lactobacillus acidophilus* is fixed at 25%; (**b1**,**b2**) show the effect of the interaction between *Lactobacillus plantarum*, *Lactobacillus brevis,* and *Lactobacillus acidophilus* on the viable bacterial number and the lactic acid production of the fermentation system when the inoculum of *Leuconostoc mesenteroides* is fixed at 25%.

To elucidate the intricate relationships between the ratios of four LAB strains and their impact on fermentation outcomes, we employed a mixture design facilitated by the Design–Expert software (v.12, NCSS, LLC, Kaysville, UT, USA). This approach yielded insightful ternary contour plots and 3D response surfaces, graphically illustrating the interplay among strains and their influence on fermentation dynamics (Figure 2). Our findings, substantiated by regression analyses (Tables S5 and S6), revealed that strain ratios significantly affect both the number of viable bacteria and lactic acid production. Notably, the interaction between *S. acidophilus* and *L*. *membranaceus* was most pronounced in enhancing bacterial activity, while the synergy between *S. acidophilus* and *L. mesenteroides* was pivotal for lactic acid yield. These interactions underscore the potential for strain-specific optimization in industrial fermentation processes.

The regression models developed from our mixture Design–Experiments not only accurately predict the fermentation outcomes but also highlight the nuanced interactions among LAB strains. The pronounced effects of strain combinations on lactic acid yield and bacterial viability are pivotal for optimizing fermentation processes in the food industry. Our findings provide a scientific basis for tailoring LAB consortia to achieve desired fermentation profiles, thereby enhancing the quality and consistency of fermented products.

The ratio of the four strains was optimized by the response surface methodology (RSM); the results showed that the interaction among the four strains of LAB was significant, and the change in the proportion of *L. mesenteroides* had the most significant impact on the number of active bacteria and the production of lactic acid in the fermentation system. The optimal mix ratio obtained is *L. mesenteroides:L. plantarum:L. brevis:S. acidophilus* = 0.313:0.00:0.160:0.527. In this optimized rationing scheme, it was predicted that the active bacteria count of the mixed fermentation system could reach 1.72424 × 10^9^ cfu/mL, and the lactic acid yield could reach 7.43171 mg/g (Table S7). To facilitate the practical operation, a validation test was conducted with the ratio of *L. mesenteroides:L. brevis:S. acidophilus* = 2:1:3. The test was repeated three times, and the average value of the measured active bacteria count of the fermentation system was 1.73 × 10^6^ cfu/mL, and the lactic acid yield was 7.42827 mg/g. The analysis revealed no significant difference between the results and the predicted values of the model, indicating that using Design-Expert to optimize the ratio of LAB in the mixture design has practical significance.

### 3.3. Determination of the Processing Technology of LAB Fermented Tea Samples

#### 3.3.1. Single Factor Test Results of the Fermentation Process

**Figure 3:**
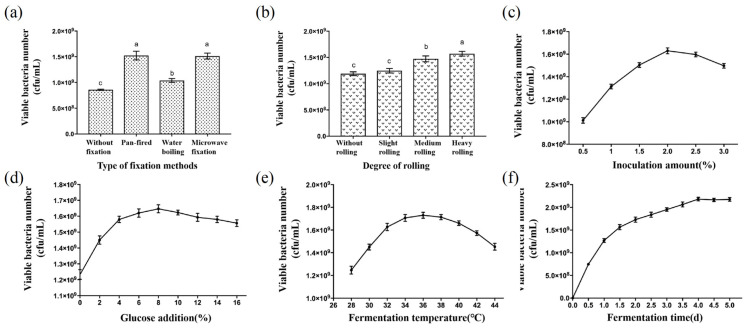
The number of viable bacteria in fermented tea is made by different factors. Note: (**a**) The number of viable bacteria in fermented tea made by different types of fixation method; (**b**) The number of viable bacteria in fermented tea produced by different degrees of rolling; (**c**) The number of viable bacteria in fermented tea made with different inoculum amounts; (**d**) The number of viable bacteria in fermented tea made with different glucose additions; (**e**) The number of viable bacteria in fermented tea made at different temperatures; (**f**) The number of viable bacteria in different days of fermented tea. Different lowercase letters mean significant differences at *p* < 0.05.

The impact of various fixation methods on the number of active bacteria in fermented tea is illustrated in Figure 3a. Fermented tea produced without fixation had the lowest count at 8.60 × 10^8^ cfu/mL, while pan-firing resulted in the highest count of 1.52 × 10^9^ cfu/mL, significantly higher than the non-fixation and boiling methods. However, there was no significant difference compared to the microwave fixation method (*p* < 0.05). Microwave fixation was selected for subsequent experiments to facilitate experimental operations.

Figure 3b shows the effect of different degrees of rolling on the number of active bacteria in fermented tea. The highest count, 1.57 × 10^9^ cfu/mL, was observed after heavy rolling, with an increase in active bacteria as the degree of rolling intensified. This indicates that the degree of rolling significantly impacts the number of active bacteria, with heavy rolling showing a notably higher count compared to the other three degrees. It is speculated that after heavy rolling, the cells of the fresh tea leaves are highly ruptured, and more internal substances of the tea leaves are released along with the tea sap. Some of the tea polyphenols are oxidized when exposed to the air, thus making the fermentation environment more suitable for the growth of lactic acid bacteria.

The influence of the inoculation amount on the active bacteria count in fermented tea is depicted in Figure 3c. The count increased with inoculum up to a certain point. Beyond 2.5% inoculum, the count decreased as the inoculum increased further. Among the six parameter points, an inoculum of 2% achieved the maximum active bacteria count of 1.63 × 10^9^ cfu/mL in the mixed bacteria fermentation system.

Figure 3d demonstrates the effect of glucose addition to the fermentation system. Glucose addition increased the LAB fermentation substrate concentration and accelerated the initial growth of LAB. With less than or equal to 8% glucose added, the active bacteria count increased with the additional amount; the count reached its maximum at 2.18 × 10^9^ cfu/mL.

#### 3.3.2. LAB Fermented Tea Process Optimized by the RSM

**Figure 4:**
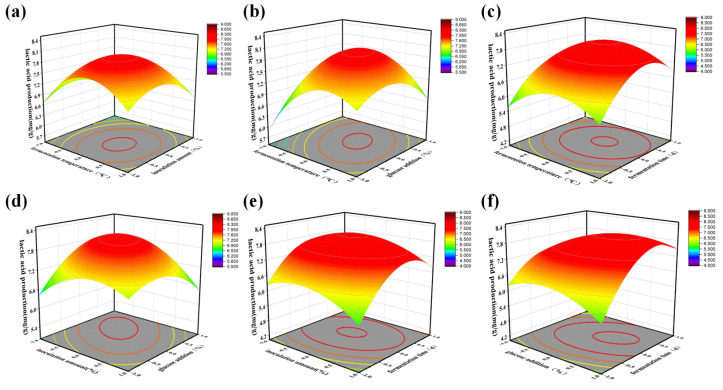
Effect of the interactions between different fermentation process factors on lactic acid production. (**a**) The effect of the interaction between the fermentation temperature and the inoculation amount; (**b**) The effect of the interaction between the fermentation temperature and glucose addition; (**c**) The effect of the interaction between the fermentation temperature and the fermentation time; (**d**) The effect of the interaction between the inoculation amount and glucose addition; (**e**) The effect of the interaction between the inoculation amount and the fermentation time; (**f**) The effect of the interaction between glucose addition and the fermentation time.

The RSM was applied to optimize the fermentation conditions (Figure 4), and ANOVA was performed to analyze the interaction between the four factors (Table S8). Results showed that the fermentation time exerted the most significant effect on the lactic acid yield, and the number of LAB had stabilized and reached as much as 10^9^ cfu/mL at 3.5 day to 4.5 day of fermentation. At this point, the lactic acid yield was improved more obviously by appropriately extending the fermentation time. The amount of lactic acid bacteria inoculation directly affects the quality of fermented products; if the inoculum is too low, the fermentation time is longer, the acid production is low, and it can be contaminated by stray bacteria [20]. After optimizing the inoculum interval to 1.5%~2.5% by single-factor test, the change in the value taken in the interval could not cause a large change in the response value. The inoculum posed the least effect on the lactic acid yield in the constructed model, probably because the bacteria have a strong growth and reproduction ability and can proliferate rapidly in a nutrient-sufficient environment, and the lactic acid yield is closely related to the number of LAB. The stable value of the number of active bacteria could be reached after 24 h with various inoculum amounts inoculated into the tea, so it was the least significant. In deviation from the conventional liquid fermentation of lactobacilli-fermented tea, studies have reported that the addition of 2% to 4% yogurt to tea extract enhances fermentation acidity and product quality, with optimal results at the 3% threshold, beyond which further increases in acidity are negligible [26]. The addition of glucose into the fermentation system can increase the fermentation substrate of LAB and promote their growth. In addition, the fermentation temperature is also a very important factor. After process optimization, the most suitable fermentation temperature was 36.5 °C, consistent with the previously established optimal fermentation temperature of 37 °C for the fermentation of Tieguanyin tea extract using lactic acid bacteria [20]. The optimum growth temperature of the strains selected in this paper varies. The optimum fermentation temperature of mixed bacteria obtained through fermentation experiments was between 34 °C and 35 °C, indicating that it is directly related to the optimum growth temperature of the fermentation strains. The process optimization was carried out with tea leaves as the substrate under the optimal ratio of loaded bacteria, so it is strongly pertinent.

Based on the conditions obtained from the response surface optimization, the best process parameters for this experiment were determined as a fermentation time of 4.005 d, a fermentation temperature of 36.54 °C, an inoculum level of 1.83%, a glucose addition of 8.88%, and a lactic acid yield of 8.12 mg/g under the optimal process. To facilitate the practical operation of the experiment, the optimal process was adjusted to a fermentation time of 4 d, a fermentation temperature of 36.5 °C, an inoculum level of 1.8%, and a glucose addition of 8.8%. Applied with the adjusted optimal process parameters, the LAB tea fermentation test was repeated three times, and their average lactic acid production was 8.08 mg/g, which was not significantly different from the model prediction value. Therefore, the optimization of the fermentation process was in line with the facts.

### 3.4. Dynamic Changes in Quality of Mixed-Bacteria Fermented Tea

#### 3.4.1. Changes of Soluble Solids before and after Fermentation

Soluble solids are one of the most basic indicators of tea quality, and their amount represents the level of tea’s substance content. The less soluble solids, the tasteless the tea soup. The soluble solids content increased slightly after fermentation, but there was no significant difference with unfermented tea (Table 2).

#### 3.4.2. Changes of Tea Polyphenols before and after Fermentation

Tea polyphenols are active in nature and undergo various forms of transformation under different processing methods. The content of tea polyphenols decreases after fermentation, presumably partly oxidized during the fermentation process and partly decomposed due to the action of LAB. No studies on the effect of LAB on tea polyphenols have been reported, and their mechanism of action is still unclear. During the fermentation of dark tea by *Scrophularia coronata*, polyphenols were converted to tea pigments and other substances mainly through three pathways: enzymatic oxidation, auto-oxidation, and hydrolysis, which may also be the reason for the decrease of tea polyphenols in this experiment [27]. In this experiment, the content of ester-type catechins in tea leaves decreased, and the non-ester-type catechins increased after fermentation (Table 3), suggesting that fermentation may have promoted the hydrolysis of ester-type catechins to non-ester-type catechins. Ester-type catechins have a strong astringent and bitter taste, while non-ester-type catechins have a more mellow taste [28,29]; the change in catechin fraction was beneficial in reducing the bitterness of tea (Table 4).

#### 3.4.3. Changes of Total Amino Acids before and after Fermentation

Amino acids are the key substances for the fresh and sweet taste of tea soup [30], among which leucine, isoleucine, phenylalanine, lysine, methionine, valine, and threonine are essential amino acids, and aspartic acid and glutamic acid are fresh amino acids. Table 3 shows the comparison of the amino acid fractions of the fermented tea sample and the control tea sample. The table shows that all essential amino acids have increased to some extent after fermentation by the *Lactobacillus* complex. While theanine was the main component of amino acids, it decreased from 5.327 mg/g to 2.835 mg/g after fermentation, which represents a decrease of 46.78%, resulting in a lower total amino acid in the fermented tea sample than the control, but the difference was not significant. The freshness of amino acid aspartic acid was originally very low, and although the content increased after fermentation, it was still low enough to have an effect on the flavor of the tea. Glutamic acid is a synthetic precursor of γ-aminobutyric acid (GABA) [31], and its content decreased after fermentation, corresponding to a significant increase in the content of GABA. GABA is a class of functional amino acids with special physiological activity, which not only participates in physiological metabolic activities in animals but also has many health benefits [32]. LAB fermentation is one of the methods to enrich GABA [33], which may be the reason for the increase in GABA content.

#### 3.4.4. Changes in Aroma Components

**Figure 5:**
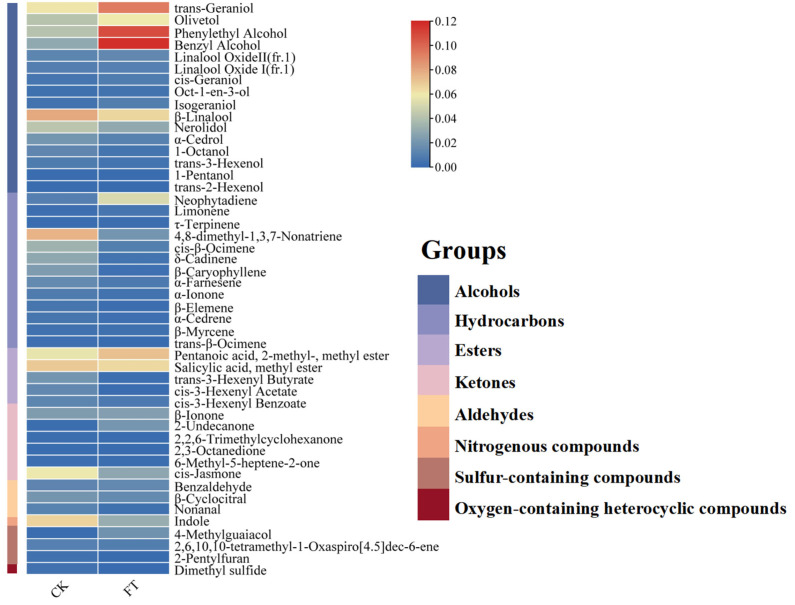
Aroma substances in samples before and after fermentation. Note: CK—Unfermented tea samples; FT—Tea fermented by LAB for five days.

Aroma is an important factor when judging the quality of tea leaves [34]. Tests showed that the aroma components of tea leaves had changed significantly after fermentation. A total of 48 volatile components were identified by the retention index and mass spectrometry. The content of 31 aroma components declined after fermentation, while the other 17 grew. These 48 components can be classified into eight categories, namely alcohols, hydrocarbons, esters, ketones, nitrogenous compounds, aldehydes, oxygen-containing heterocyclic compounds, and sulfur-containing compounds (Figure 5). After fermentation, except for alcohols, the content of the other seven kinds of aroma substances decreased, with alcohols being the main components of the volatile component, accounting for 54.63%. The top three aroma components in the fermented tea samples were benzyl alcohol, phenethyl alcohol, and geraniol; all have special floral and fruit aromas [35]. The content of 4-methyl guaiacol increased the most after fermentation, about ten times compared with the control. 4-Methyl guaiacol was detected in tea in 1987 and exists in some post-fermented teas. It is also a representative aroma component in Japanese pickled tea.

*β*-Linalool was the most abundant aroma component in the control tea samples, amounting to 7.96%. It decreased in content after fermentation, but the decline was small. Indole generally exists in natural flower oil. Compared with the control, the content of indole in fermented tea decreased by about 50%. Nerolidol has a slightly mellow, woody aroma, which tends to decline during fermentation. Methyl salicylate is an aroma component with a special smell of herbal medicine, and microbial fermentation has little effect on it.

## 4. Conclusions

In this study, compound LAB was used to ferment summer and autumn green tea for the first time, and the main quality components of tea samples before and after fermentation were determined and analyzed. Featuring a great antibacterial effect, tea polyphenols are high in summer and autumn tea, hence it is important to screen the LAB suitable for summer and autumn tea fermentation. Four kinds of such LAB were screened out in the study, which are *L. mesenteroides*, *L. plantarum*, *L. brevis*, and *S. acidophilus*. The optimized strain ratio of the mixed design was *L*. *membranaceus*:*L. brevis:S*. *acidophilus* = 2:1:3. Through the single factor test, it was determined that the best process for the production of mixed-bacteria fermented tea leaves is microwave fixation and heavy rolling. The best parameters of the fermentation process were a 4-day fermentation time, a 36.5 °C fermentation temperature, a 1.8% inoculum, and an 8.8% glucose addition. Compared with unfermented tea samples (CK), the content of tea polyphenols and ester-type catechins, relating to bitterness and astringency, decreased significantly in fermented tea (FT), while the content of mellower non-ester-type catechins increased. The total amount of free amino acids did not change much, whereas theanine in the amino acid components reduced significantly, and the content of GABA, which has multiple health effects, considerably rose. Among the aroma components, FT contains more alcohol, emitting a better floral and fruit aroma. This study shows that LAB fermentation can reduce the bitterness and astringency of summer-autumn tea, providing new insights into the utilization of tea production in these two seasons, which can improve their utilization to a certain extent and increase the income of tea farmers.

## Figures and Tables

**Table 1:** The active bacteria and lactic acid yield of six strains after 24 h incubation.

Strains	Active Bacteria Count(cfu/mL)	Lactic Acid Yield(mg/g)
*Lactobacillus bulgaricus*	8.61 × 10^7^ e	6.09 ± 0.06 d
*Lactobacillus brevis*	4.63 × 10^8^ d	6.50 ± 0.03 c
*Lactobacillus plantarum*	6.11 × 10^8^ c	6.54 ± 0.05 c
*Streptococcus thermophilus*	7.24 × 10^7^ e	5.99 ± 0.06 e
*Leuconostoc mesenteroides*	6.82 × 10^7^ b	6.78 ± 0.06 b

Note: Values are mean ± SD. Different lowercase letters mean significant differences at *p* < 0.05.

**Table 2:** Dynamic changes of major biochemical components in tea during fermentation.

Fermentation Time (day)	Main Biochemical Components (%)			
	Soluble Solids	Tea Polyphenols	Total Free Amino Acids	Soluble Sugar
0	36.53 ± 0.16 a	24.08 ± 0.13 a	1.95 ± 0.03 a	4.23 ± 0.03 a
1	34.46 ± 0.16 d	21.52 ± 0.18 b	1.78 ± 0.03 c	3.11 ± 0.10 b
2	35.20 ± 0.13 c	19.54 ± 0.09 c	1.72 ± 0.03 d	2.47 ± 0.03 c
3	36.09 ± 0.11 b	17.75 ± 0.14 d	1.80 ± 0.03 c	1.81 ± 0.02 d
4	37.42 ± 0.05 a	17.01 ± 0.03 e	1.84 ± 0.02 bc	1.29 ± 0.03
5	37.40 ± 0.16 a	16.54 ± 0.19 f	1.89 ± 0.03 ab	1.22 ± 0.02 e

Note: Values are mean ± SD. All units represent percentages of dry weight. Different lowercase letters mean significant differences at *p* < 0.05.

**Table 3:** Content of major biochemical components in CK and FT.

Groups	Groups	CK	FT
Catechins (mg/g)	Galloylated catechins	56.84	79.88
	Non-Galloylated catechins	59.24	24.82
	Total catechins	116.08	104.7
Amino acids (mg/g)	Theanine	5.327	2.835
	Leu	0.073	0.105
	Iie	0.042	0.064
	Phe	0.085	0.152
	Lys	0.038	0.186
	Met	0.139	0.178
	Val	0.147	0.163
	Thr	0.087	0.102
	Asn	0.008	0.019
	Glu	0.342	0.298
	GABA	0.051	0.126
	Ser	0.418	0.334
	Gly	0.469	0.425
	His	0.126	0.112
	Arg	0.063	0.149
	Ala	0.088	0.132
	Pro	0.315	0.478
	Cys	0.847	1.157
	Tyr	0.051	0.134
	Total	8.716	8.278

Note: CK—Unfermented tea samples; FT—Tea fermented by LAB for five days.

**Table 4:** Dynamic changes of catechin in tea during fermentation.

Fermentation Time (day)	GA	C	EC	EGC	ECG	EGCG	Total Catechins
0	1.67 ± 0.04 f	7.86 ± 0.04 e	9.56 ± 0.06 e	39.26 ± 0.06 f	8.68 ± 0.05 a	49.05 ± 0.05 a	116.08 ± 0.16 a
1	2.47 ± 0.07 e	7.17 ± 0.04 f	10.15 ± 0.10 d	40.21 ± 0.08 e	8.36 ± 0.05 a	46.23 ± 0.05 b	114.58 ± 0.09 b
2	4.02 ± 0.04 d	8.83 ± 0.07 d	13.72 ± 0.10 c	42.66 ± 0.25 d	7.89 ± 0.04 b	37.57 ± 0.04 c	114.70 ± 0.31 b
3	4.85 ± 0.05 c	10.17 ± 0.07 c	15.36 ± 0.08 b	44.75 ± 0.05 c	7.29 ± 0.20 c	26.12 ± 0.08 d	108.54 ± 0.15 c
4	5.35 ± 0.09 b	11.74 ± 0.05 b	18.43 ± 0.27 a	47.41 ± 0.05 b	7.00 ± 0.23 c	18.38 ± 0.05 e	108.41 ± 0.20 c
5	6.15 ± 0.07 a	13.10 ± 0.10 a	18.17 ± 0.10 a	48.45 ± 0.05 a	6.50 ± 0.30 d	12.33 ± 0.05	104.70 ± 0.33 d

Note: Values are mean ± SD. All units are mg/g. Different lowercase letters mean significant differences at *p* < 0.05.

## Data Availability

All research data can be found in this manuscript and the Supplementary Materials.

## Supplementary Materials

The following supporting information can be downloaded at https://www.mdpi.com/article/10.3390/foods13193126/s1, Table S1: The factors and levels of the LAB mixture experiment; Table S2: The technological level design of the single-factor experiment of the fermentation process; Table S3: The factors and levels of the response surface test; Table S4: Mixture Design–Experiment table; Table S5: The analysis results of the regression model of the lactic acid production; Table S6: The analysis results of the regression model of viable bacterial count; Table S7: Optimized ratio scheme and response values; Table S8: Content of major biochemical components in CK and FT.
